# The Role of Central and Peripheral Brain-Derived Neurotrophic Factor (BDNF) as a Biomarker of Anorexia Nervosa Reconceptualized as a Metabo-Psychiatric Disorder

**DOI:** 10.3390/nu16162617

**Published:** 2024-08-08

**Authors:** Jingxian Cao, Philip Gorwood, Nicolas Ramoz, Odile Viltart

**Affiliations:** 1 Institute of Psychiatry and Neuroscience of Paris (IPNP), Université Paris Cité, INSERM UMR-S 1266, F-75014 Paris, Franceodile.viltart@inserm.fr (O.V.); 2 GHU Paris Psychiatrie et Neurosciences, CMME, Hôpital Sainte Anne, F-75014 Paris, France; 3 SCALab Laboratory, PsySEF Faculty, Université de Lille, UMR CNRS 9193, F-59650 Villeneuve d’Ascq, France

**Keywords:** anorexia nervosa, brain-derived neurotrophic factor (BDNF), eating disorders, val66met polymorphism, metabolism

## Abstract

Neurotrophic factors play pivotal roles in shaping brain development and function, with brain-derived neurotrophic factor (BDNF) emerging as a key regulator in various physiological processes. This review explores the intricate relationship between BDNF and anorexia nervosa (AN), a complex psychiatric disorder characterized by disordered eating behaviors and severe medical consequences. Beginning with an overview of BDNF’s fundamental functions in neurodevelopment and synaptic plasticity, the review delves into recent clinical and preclinical evidence implicating BDNF in the pathophysiology of AN. Specifically, it examines the impact of BDNF polymorphisms, such as the Val66Met variant, on AN susceptibility, prognosis, and treatment response. Furthermore, the review discusses the interplay between BDNF and stress-related mood disorders, shedding light on the mechanisms underlying AN vulnerability to stress events. Additionally, it explores the involvement of BDNF in metabolic regulation, highlighting its potential implications for understanding the metabolic disturbances observed in AN. Through a comprehensive analysis of clinical data and animal studies, the review elucidates the nuanced role of BDNF in AN etiology and prognosis, emphasizing its potential as a diagnostic and prognostic biomarker. Finally, the review discusses limitations and future directions in BDNF research, underscoring the need for further investigations to elucidate the complex interplay between BDNF signaling and AN pathology.

## 1. AN: A Metabo-Psychiatric Disorder with Complex Etiology

Human brain-derived neurotrophic factor (BDNF), a member of the neurotrophin family, has long been recognized for its regulatory roles in crucial functions, such as neuron survival, differentiation, and specification. In the mature nervous system, BDNF modulates synaptic transmission and axonal morphology, ultimately affecting behavior, learning, memory and, in particular, feeding behavior [1]. Alterations in BDNF pathways have been described in several psychiatric disorders. In recent decades, its intricate involvement has extended to eating disorders, with compelling evidence linking BDNF to several disorders including anorexia nervosa (AN) [2].

After a short description of the role and functions of BDNF, our review will update recent clinical and preclinical data showing how BDNF can be a key biomarker in the physiopathology of AN.

AN is a complex psychiatric disorder characterized by voluntary dietary limitation and excessive physical activity due to the patient’s fear of weight gain and desire to lose body weight although underweight, according to DSM-5 [3]. AN encompasses two main subtypes: the restrictive type (AN-R), characterized by severe food restriction, and the binge–purge type (AN-BP), marked by episodes of binge eating followed by purging behaviors [4]. The female-to-male ratio of AN is around 10/1 to 15/1, young women being the most vulnerable [5,6]. Anorexia nervosa presents a substantial threat, bearing the highest mortality rate among psychiatric disorders. Patients often persist in food restriction despite reaching dangerously low body mass index (BMI), risking severe malnutrition and medical complications, including organ failure [7,8,9]. In addition, the high comorbidity with numerous psychiatric disorders such as major depressive disorder (MDD) and anxiety disorder (AD) highly increases the risk of patients to commit suicide [10,11,12]. Indeed, AN is notoriously difficult to treat, with a complex chronicity and a relapse rate of up to 50% in the first year, and with more than 20% of patients eventually experiencing recurrent patterns of remission and relapse [6,13,14,15]. Currently, the only proven treatment for AN remains re-feeding strategies combined with psychotherapy, as there are still no effective pharmacological treatments [16].

**Figure 1:**
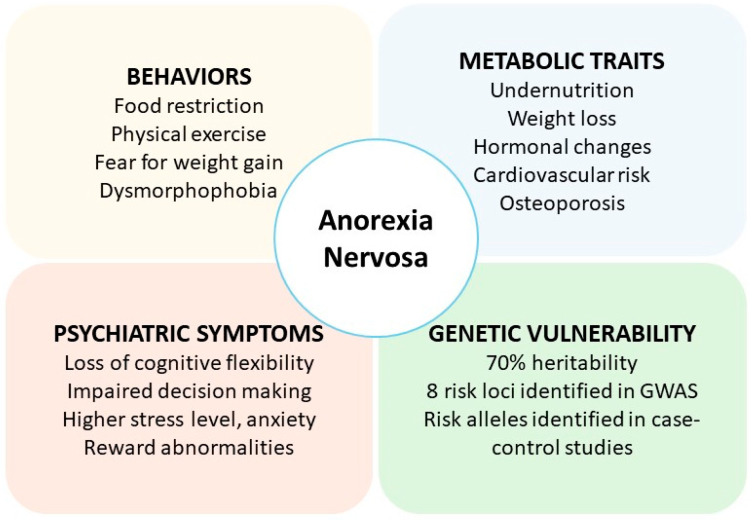
Multifaceted symptoms of anorexia nervosa.

Recently, AN has been reconceptualized as a metabo-psychiatric disorder according to a genome-wide association study (GWAS), which provides evidence that various genetic factors are involved in the etiology of AN, related to both neuro-psychiatric factors and to anthropometric and metabolic pathways [17]. In support of this notion, substantial evidence found in familial associations studies highlights the high heritability of AN (up to 70%) [18,19,20]. Furthermore, a large-scale GWAS identified eight risk loci associated with AN [17]. Case-control studies have also pointed out other genetic risk factors for AN, such as 5-hydroxytryptamine receptor 2A (HTR2A), serotonin transporter (5-HTT), estrogen receptor 1 (ESR1), or BDNF [21,22,23]. More particularly for the latter, the rs6265 polymorphism (or Val66Met) is clearly associated with AN symptomatology [24,25], while BDNF has been shown to be closely involved in both mood and metabolic regulation, the two main physiological aspects of AN (Figure 1). 

## 2. Brain-Derived Neurotrophic Factor and Val66Met Polymorphism

**Figure 2:**
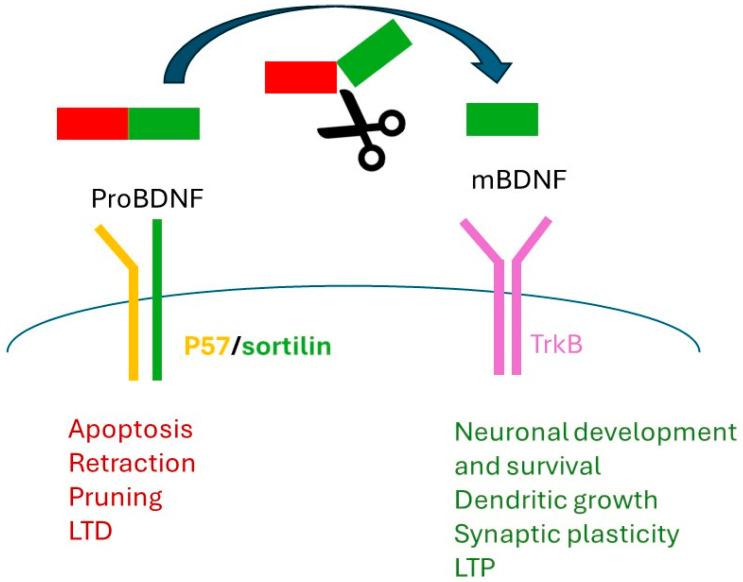
Signaling Pathways of BDNF Protein. This figure illustrates the primary signaling pathways activated by BDNF binding to its receptors, TrkB and p75NTR. Upon mBDNF binding, TrkB receptors initiate downstream signaling cascades and promote neuronal survival, synaptic plasticity, synaptogenesis, and long-term potentiation (LTP). The interaction of pro-BDNF with its receptor p75NTR is involved in apoptosis, pruning and retraction of synaptogenesis, and long-term depression (LTD).

The human BDNF gene is located on chromosome 11, region p13–14 and spans approximately 70 kb [26]. The BDNF gene encodes a neuropeptide that is first produced in neurons as pro-BDNF, then cleaved to mature BDNF (mBDNF), and finally secreted into the synapse to bind to the tropomyosin receptor kinase B (TrkB) (NTRK2/TrkB gene) on the postsynaptic membrane [27,28,29,30]. BDNF belongs to the neurotrophin family [31]. Its signaling through the TrkB receptor is essential for neuronal survival, neuronal development, synaptogenesis, and long-term potentiation [32,33,34,35]. Pro-BDNF has a different signaling profile from BDNF, as it can bind directly to the p75 neurotrophin receptor (p75NTR), which promotes neuronal apoptosis and synaptic pruning [36,37,38]. The BDNF gene is essential for early survival, as total knockout of the BDNF gene in mice results in severe malformation and death in the first postnatal days [39] (Figure 2).

Several polymorphisms (SNP) have been identified in the human BDNF gene, of which rs6265 (Val66Met) is one of the most studied. Val66Met is a functional variant of human BDNF in which the 66th amino acid, a valine, is substituted by a methionine [40]. 

**Figure 3:**
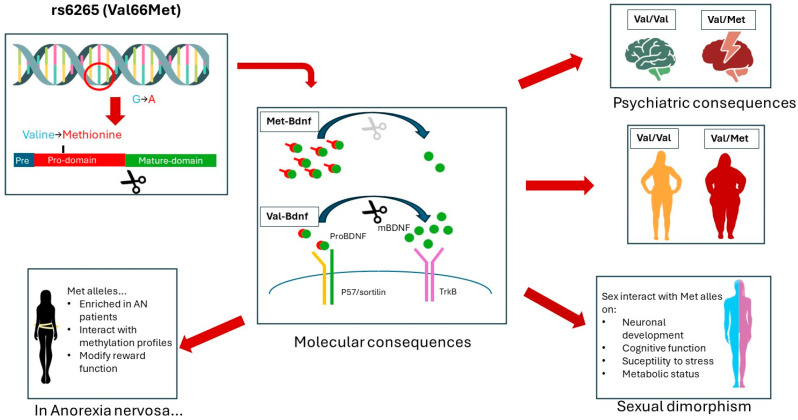
Influence of Val66Met polymorphism on molecular mechanisms and physiological outcomes related with AN. The molecular level effects of the Val66Met polymorphism include alterations in BDNF signaling towards p57 receptor, and a decrease in the availability of mBDNF. At the physiological level, human Met carriers are more likely to develop psychiatric vulnerabilities to environmental stress and metabolic diseases such as obesity. Certain influences of the Val66Met polymorphism exhibit sexual dimorphism. In the context of AN, Met alleles are currently of interest.

The Val66Met polymorphism in the BDNF gene results in significant changes in BDNF signaling, affecting multiple aspects of neuronal function and behavior. Mechanistically, Met carriers exhibit attenuated BDNF signaling due to impaired epigenetic regulation, disrupted dendritic trafficking of BDNF mRNA, and reduced availability of mature BDNF and its secretion [41,42,43,44]. These alterations result in lower basal BDNF levels and TrkB signaling, impaired neurogenesis, and cognitive deficits observed in both animal models and human studies [41,45,46,47,48,49] (Figure 3).

## 3. BDNF and AN: Clinical Data

The association between the Val66Met polymorphism and AN has been investigated in various studies, with inconsistent findings. Indeed, a meta-analysis encompassing nine studies and involving a total of 2767 cases and 3322 controls found no clear association between the Val66Met polymorphism and the onset of AN [50], while certain demographic groups, such as individuals of Spanish descent, have exhibited an association between the Met variant and restrictive AN, along with a correlation between the number of Met alleles and the minimum body mass index (BMI). Recently, a genome-wide association study (GWAS) found no significant associations between the BDNF gene and AN [17]. 

These inconsistencies reflect differences between populations and methods. Factors such as sample size, patient demographics, and study design may indeed contribute to the variability of results. Nevertheless, recent studies have suggested that the Met variant may increase the risk of developing AN, particularly in individuals with comorbid borderline personality disorder [51]. The Val66Met polymorphism was also associated with an increased reward value attributed to images depicting starvation [25] in patients with restrictive AN, supporting several psychological symptoms or phenotypes associated with genetic of BDNF (Figure 3). For AN-BP, studies have not found significant associations with the Val66Met polymorphism [52]. Other BDNF haplotypes have been implicated in AN and related eating disorders, such as bulimia nervosa [53,54]. However, further research is needed to elucidate the precise role of BDNF and its receptor genes in the etiology and symptomatology of AN.

Conflicting results were also observed for circulating BDNF levels. While some reported decreased BDNF levels in patients with AN [50,55], other studies report higher plasmatic BDNF levels in patients with AN, particularly those carrying the Met variant [24,54]. The increase in circulating BDNF remained at supranormal levels up to 2.5 years after first hospitalization [56]. Despite some discrepancies concerning the variation in BNDF, a certain number of studies have linked the variation in BDNF signaling and specific symptoms of AN. For instance, reduced levels of circulating BDNF were observed in patients with AN with very low body mass index upon admission, while weight rehabilitation subsequently restored BDNF plasma levels [55]. Though BDNF levels in the cortex or serum have been found to positively correlate with cognitive flexibility in various animal models and human subjects [57,58,59], there is no correlation in patients with AN between serum BDNF levels and impaired cognitive flexibility [60]. Thus, limited evidence exists regarding the relationship between the Val66Met polymorphism and anthropometric and psychological parameters in AN patients [61]. 

These data point out a complex and nuanced relationship between the expression of BDNF and AN pathology, contingent upon the presence or absence of the variant, the subtype of AN, and the stage of the disorder. A better investigation of this biomarker could unveil significant implications for comprehending the psychiatric manifestations linked to the disorder [61].

## 4. BDNF and AN: Inputs from Animal Models

While no animal model can fully capture all aspects of AN, due to its complexity both at metabolic and psychological levels, the rodent models currently used in research mimic specific facets of the disorder. These models serve to simulate certain behaviors or metabolic conditions observed in AN rather than replicating the disorder in its entirety. For instance, short-term food restriction models, such as the activity-based anorexia (ABA) model, aim to replicate aspects of the restrictive eating behavior seen in AN [62]. Meanwhile, chronic food restriction models, such as the food restriction with running wheels (FRW) model, focus on mimicking the long-term consequences of caloric deprivation often associated with AN [63]. 

**Figure 4:**
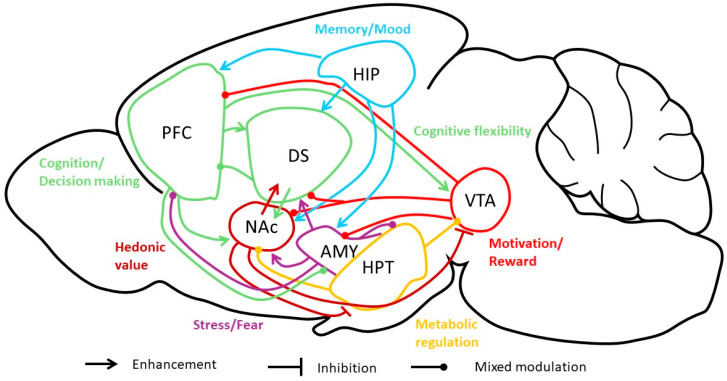
Brain circuits implicated in anorexia symptomatology. PFC: prefrontal cortex. DS: dorsal striatum. NAc: nucleus accumbens. AMY: amygdala. HIP: hippocampus. VTA: ventral tegmental area. HPT: hypothalamus [96,97,98].

Examining the BDNF expression profile in brain regions of rodents subjected to the ABA model reveals interesting dynamics (Table 1). During scheduled feeding in the ABA model, rodents show increased BDNF levels in the hippocampus but decreased levels in the medial prefrontal cortex (mPFC), contributing to anorexia-like behaviors via alterations in the brain’s reward circuitry [64,65]. In an ABA experiment, it was demonstrated that the use of a running wheel increases BDNF expression in the ventral tegmental area (VTA), while BDNF levels in the hippocampus or nucleus accumbens (NAc) were not affected by either wheel running or food restriction [65]. Furthermore, a recent study highlighted a persistent downregulation of BDNF signaling in the amygdala of ABA-exposed rodents, despite an increase in mature BDNF protein levels, underscoring the complex neurobiological alterations induced by food restriction [66]. The mPFC has a critical role in executive functions such as cognitive control and decision making, the hippocampus is more involved in memory formation and emotional regulation, while the VTA is a core structure in reward processing (Figure 4). Interestingly, these three structures were implicated in AN onset [67,68,69,70,71,72]. Alterations of BDNF expression within these brain regions in response to food restriction provide compelling evidence supporting the hypothesis that AN initiation may involve aberrant BDNF signaling. 

**Table 1:** Regional modification of BDNF under metabolic or psychological stress.

Tissue	Gene	Calory Restrictions (Chronic)		Fasting (Acute)		Physical Activities		High Fat Diet/Obesity		Chronic Stress		Acute Stress	
		Change	Species/Sex	Change	Species/Sex	Change	Species/Sex	Change	Species/Sex	Change	Species/Sex	Change	Species/Sex
PFC	BDNF	Ns Prot	Rat ♂ [73,74]			↑ mRNA	Mice ♂ [75]	↓ Prot	Rats ♂ [76]	↓ mRNA	Rat ♂ [77]	↓ mRNA	Rat ♂ [77]
		↑ Prot	Rat ♀♂ [78,79]									↑ Prot	Rat ♂ [80]
Hippocampus	BDNF	↑ Prot	Rat ♀♂ [73,78,79]	↓ Cell Nb	Mice ♂ [81]	Ns mRNA	Mice [75]	↓ Prot	Rats ♂ [76]				
		↓ Cell Nb	Rat ♂ [82]	↑ Prot	Mice ♂ [83]			↓ mRNA	Mice ♂ [84]				
	TrkB	Ns Cell Nb	Rat ♂ [82]					↓ mRNA	Mice, ♂ [84]			Ns Prot	Rat ♂ [80]
Hypothalamus	BDNF							↓ mRNA	Mice ♂, Rat ♀♂ [84,85]	↑ mRNA, prot	Rat ♂ [86]	↑ mRNA	Rat ♂ [87]
	TrkB							Ns mRNA	Mice ♂ [84]				
DS	BDNF					↑ Prot	Mice ♂ [88]			↑ mRNA,prot	Mice ♂ [89]		
NAc	BDNF	Ns Prot	Rat ♂ [74]							↑ mRNA	Human ♀♂ [90]	↑ Prot	Rat ♂ [91]
	TrkB											↑ Prot	Rat ♂ [91]
VTA	BDNF	Ns mRNA, prot	Rat ♂ [74]									↑ Prot	Rat ♂ [91]
	TrkB	↓ Prot	Rat ♂ [74]									↑ Prot	Rat ♂ [91]
Amygdala	BDNF	↑ Prot	Rat ♂ [73]	↓ mRNA	Rats ♀ (ABA) [66]					↓ mRNA, prot	Rat ♂ [92]		
Serum/Plasma	BDNF	Ns Prot	Rat ♂ [79]			↑ Prot	Human ♂ [93]	↓ Prot	Human ♀ [94]	↑ Prot	Human ♂ [95]	↑ Prot	Human ♂ [95]

Ns: no significant change. ↑: increases. ↓: decreases. Prot: protein. Nb: number. ♂: male. ♀: female.

In the ABA model, vulnerable mice exhibit maladaptive behaviors under food restriction, including increased running activities, reduced food intake, and excessive weight loss, resulting in an anorexia-like phenotype [99]. The Val66Met polymorphism does not appear to influence the manifestation of vulnerability in ABA rats [100]. Although knock-in mice with the Met variant display higher baseline anxiety, both WT and Met mice exhibit similar levels of anxiety under the ABA protocol [101]. However, in a chronic food restriction protocol, Met mice exposed to adolescent social stress show increased metabolic vulnerability and higher anxiety levels compared to WT littermates, whereas exposure to social stress during adulthood does not result in a significant difference [102], supporting the idea that the Val66Met polymorphism may create a genetic predisposition, which, when combined with exposure to juvenile social stress and calories restriction, increases the likelihood of the onset of anorexia-like behaviors. This elucidates the intricate interplay between genetic predisposition and environmental stressors, underscoring the complexity of AN etiology and may provide valuable insights for the development of targeted interventions or therapeutic strategies.

## 5. BDNF and Stress-Related Mood Disorders

It is important to recognize that AN is closely associated with stress, as stress can increase vulnerability to the disorder [103,104]. 

In the context of stress-related mood disorders, such as depression and anxiety, environmental stress significantly affects BDNF expression in various brain regions (Table 1). Studies in rodent models indicate that exposure to environmental stressors decreases BDNF mRNA expression in key brain regions implicated in mood regulation, including the hippocampus, prefrontal cortex, and amygdala [92,105,106,107]. The downregulation of BDNF mRNA in these regions is associated with the manifestation of depressive-like behaviors and increased susceptibility to stress-induced mood disorders, including decreased social interaction, heightened behavioral despair, dysphoria, and elevated anxiety levels [105,106,107]. 

Interestingly, the effects of BDNF on mood regulation vary depending on the brain region. Indeed, BDNF expression in the hippocampus is typically associated with an antidepressant effect. On the contrary, BDNF signaling in the dopamine mesocorticolimbic circuit appears to have a depressive effect, as both BDNF infusion and TrkB overexpression induce shorter latency to immobility in forced swimming tests, while BDNF KO or a reduction in BDNF signaling, via a dominant negative receptor, has opposite effects [89,108]. Increasing BDNF levels in the NAc, VTA, and dorsal striatum (DS)—a critical region for cognitive flexibility [90] (Figure 4)—contributes to the development of susceptibility to depressive-like phenotypes under conditions of repeated aggression stress [109,110,111] (Table 1). Conversely, a reduction in BDNF levels within these areas has been associated with an antidepressant effect [112]. In humans, post-mortem studies of individuals with major depressive disorder who died by suicide have further highlighted region-specific alterations in BDNF expression, with reduced hippocampal BDNF but increased BDNF levels in the NAc [111,113], suggesting a region-specific dysregulation of BDNF expression in relation to mood disorder. Such results mirror observations from animal studies and offer valuable insights into the nuanced role of BDNF signaling across various brain circuits.

The Val66Met polymorphism of the BDNF gene has been implicated in increasing genetic vulnerability to mood disorders in response to environmental stimuli. In adolescent social stressed mice, the knock-in of the human Val66Met variant increases the likelihood and severity of anorexic behavior induced by caloric restriction [102]. In addition, Met-carrying mice tend to exhibit higher adrenal responses and experience more behavioral impacts after stress exposure, including decreased social interaction, impaired novelty exploration, and higher anxiety levels [114] (Figure 3).

In human studies, the Met variant interacts with stressful life events and childhood adversity, increasing the risk of developing depression-like symptoms, neuroticism, subclinical psychotic experiences, as well as suicidal behaviors [115,116,117,118]. These interactions are often associated with corresponding alterations in brain regions such as the hippocampus and lateral prefrontal cortex, as revealed by structural magnetic resonance imaging (MRI) [119]. However, conflicting findings have also been reported, with some studies suggesting that Met carriers show reduced vulnerability to high stress and reduced sensitivity to the benefits of stress reduction [120,121]. Specifically, male Met carriers have lower cortisol responses under stress, whereas female Met carriers have higher cortisol responses under stress [122,123]. These findings highlight the complex interplay between genetic factors, stress exposure, and neurobiological mechanisms in shaping vulnerability to mood disorders. Such intricate relationships may have implications for understanding the pathophysiology of AN, particularly given its significant prevalence among females.

Collectively, these findings suggest that BDNF plays a critical role in determining vulnerability/resilience to environmental stress, with specific effects observed in different brain regions. Understanding the nuanced interactions between BDNF and environmental stressors may provide valuable insights into the pathophysiology of stress-related mood disorders, particularly associated with AN, and inform the development of targeted therapeutic interventions.

## 6. BDNF and Metabolism

The metabolic consequences of chronic starvation in individuals with AN have been well established through metabolomic studies, shedding light on the intricate relationship between metabolic dysregulation and the maintenance of the disorder [124]. Accordingly, further investigation into the metabolic role of BDNF may provide valuable insights into its potential as a biomarker for AN.

In addition to its classical roles in neuronal protection, synaptogenesis, and mood regulation, recent studies have revealed a central involvement of BDNF in metabolic regulation. Animal studies have shown that BDNF exerts its metabolic effects via hypothalamic signaling pathways. For example, targeted deletion of the BDNF gene in the paraventricular nucleus (PVN), the principal endocrinal control center in metabolic homeostasis [125], leads to hyperphagia and an obese phenotype in animals, accompanied by decreased locomotor activity and reduced thermogenesis [126]. Conversely, activation of the BDNF gene or central administration of the BDNF protein in the PVN produces opposite effects, suggesting a direct influence of PVN BDNF on hypothalamic neurons and subsequent metabolic processes [127,128,129]. 

The arcuate nucleus (ARC) in the hypothalamus is primarily composed of two groups of neurons: pro-opiomelanocortin (POMC) neurons and agouti-related peptide (AgRP)/neuropeptide Y (NPY) neurons [125]. POMC neurons are known to promote satiety and reduce food intake, while AgRP/NPY neurons stimulate appetite and increase food consumption [125]. Both types of neurons project to the paraventricular nucleus (PVN) and other hypothalamic regions, playing crucial roles in the regulation of energy balance and food ingestion [125]. A small fraction of POMC and AgRP/NPY neurons in the ARC were found to express the TrkB receptor [130]. Suppression of BDNF mRNA in the ARC leads to severe hyperphagic obesity, accompanied by decreased axonal projections from the ARC to the PVN, further demonstrating the importance of BDNF signaling in central metabolic regulation [130].

Furthermore, the effects of BDNF on metabolism extend beyond the confines of the brain, as peripheral tissues also contribute significantly to metabolic regulation and interact with central BDNF signaling. Peripheral expression of the BDNF gene is widespread in various tissues, including skeletal and smooth muscle, adipose tissue, liver, spleen, and others [131,132,133,134,135,136]. These peripheral sources of BDNF modulate metabolic processes in a complex manner.

For example, in the liver, BDNF administration activates catabolic pathways, including fatty acid oxidation and glycogen synthesis [137,138,139]. Adipose tissue-derived BDNF also plays a crucial role in metabolism by regulating local processes in white adipose tissue, such as sympathetic innervation, mitochondrial function, and susceptibility to obesity-related challenges [136,140,141]. Similarly, skeletal muscles secrete BDNF in response to contraction and fasting, thereby contributing to glucose and lipid metabolism [142,143,144,145,146]. 

Concerning the Val66Met polymorphism, numerous studies demonstrated its involvement in body weight regulation, with distinct associations in both animal and human studies. Animal models, in particular homozygous knock-in BDNF met/met mice, showed a phenotype characterized by hyperphagia, obesity, and altered inflammatory profiles in addition to reduced hypothalamic BDNF expression [48,147]. Conversely, human genotype–phenotype association studies suggest that Met-Met individuals tend to have lower body mass index compared to Val-Met or Val-Val individuals [148,149,150,151,152,153]. Furthermore, while the Met variant is not associated with serum BDNF levels, it correlates with reduced BDNF gene expression in muscle tissue, as demonstrated by human biopsy analysis [154,155,156] (Figure 3). 

These findings underscore the challenge of translating observations from human genetic variants to animal models and highlight potential differences in the regulation of BDNF signaling between species. Notably, while AN primarily involves metabolic challenges due to long-term food restriction and low body weight, research on the association of the Val66Met polymorphism with metabolism has primarily focused on obesity, with limited exploration in the context of chronic calorie restriction.

## 7. Discussion

### 7.1. Role of BDNF in AN Etiology and Prognosis

Despite the limited direct evidence linking the Met variant to AN, current evidence on interrelated BDNF polymorphisms and altered BDNF levels suggests that BDNF is a plausible candidate for the etiology of AN. The paradoxical circulating BDNF levels in AN patients underscore the complexity of this disorder and highlight the challenges in under-standing its underlying mechanisms. 

It is important to note that AN encompasses two primary subtypes: restrictive and binge–purge. These subtypes can exhibit different clinical and biological characteristics, potentially leading to varying BDNF levels and responses. Research suggests that the AN-R may have different metabolic and neurobiological profiles compared to AN-BP [154,155]. Therefore, studies examining AN as a single category may yield distorted results due to this heterogeneity. Recognizing these differences is crucial for accurately interpreting findings and developing subtype-specific biomarkers and treatments.

While animal studies clearly demonstrate the anorexigenic effects of BDNF, extrapolating its role and changes in plasma levels in patients is challenging. Several findings indicate that BDNF levels are not only influenced by acute nutritional status but may also have long-term alterations in individuals with a history of AN [56,60]. Hypothetically, persistent changes in BDNF signaling post-BMI recovery could underlie an increased risk of relapse, emphasizing the importance of considering long-term effects in the study of BDNF and AN. This aspect warrants further investigation to enhance our understanding of the relationship between BDNF and nutritional status and to explore the potential of BDNF as a biomarker for the prognosis of AN.

### 7.2. Exploring the Biomarker Potential of Circulating BDNF

Numerous studies have investigated the potential of circulating BDNF as a biomarker for various diseases. Subsequently, circulating BDNF levels have been found to correspond to psychiatric status, with interactions with genotype [108,112,156]. Changes in plasma BDNF levels vary, showing either increases or decreases in different psychiatric disorders [157,158,159,160,161]. However, as mentioned above, the results in individuals with AN are paradoxical.

Circulating BDNF may also serve as a marker for the psychiatric prognostic status of AN patients, given the propensity of AN patients to have psychiatric symptoms that implicate BDNF. For example, AN patients often exhibit reward abnormalities, and BDNF has been implicated in reward dependence [162,163]. The prevalence of childhood adversity and stressful life events in AN patient further supports the role of BDNF as an intermediary factor in the relationship between life stress and psychiatric disorders [108,112,156,164]. 

### 7.3. Metabolic Implications and Sexual Dimorphism

Exploration of the relationship between circulating BDNF and metabolic status has predominantly focused on obesity and responses to dietary interventions such as chronic calorie restriction and intermittent fasting, with somewhat inconclusive results [144,165,166]. Ethical considerations limit studies into the effects of long-term food restriction on circulating BDNF levels in healthy subjects. Nevertheless, existing studies suggest that chronic calorie-restricted diets may increase serum BDNF levels in overweight populations and negatively correlate with body weight [167]. 

AN has a strong female sexual dimorphism that cannot yet be explained by sociological factors. Female rats experiencing maternal separation show decreased anxiety behavior under the ABA model, suggesting a unique interaction between stress and dieting that is also highly sexually dimorphic [168]. The effects of BDNF on psychiatric traits and metabolism often also exhibit sexual polymorphism, highlighting additional features shared with the etiology of AN and further strengthening its potential implications [142,169,170,171].

Recent years have seen an increasing recognition of AN in males, who now represent a significant subset of those affected by the disorder. While men with AN often present with similar core symptoms as women, such as restrictive eating and intense fear of gaining weight, they may place more emphasis on muscularity and leanness rather than thinness alone [172,173]. This gender-specific focus on body image can lead to behaviors like excessive exercise, which may sometimes be overlooked in diagnostic assessments [172,173]. Male patients with anorexia nervosa often experience more significant weight loss prior to treatment and require longer durations of hospitalization for weight restoration compared to their female counterparts [174]. Psychiatric comorbidities in males with AN, such as lower rates of specific phobia, also differ from those typically observed in females [175], potentially influencing treatment outcomes. 

Given these differences, there is a critical need for gender-specific research on BDNF in AN. Understanding the role of BDNF in male AN patients could lead to the development of more effective, personalized treatment strategies.

### 7.4. The Paradox of Physical Activity

Physical activity plays a significant role in the regulation of BDNF levels, which is particularly relevant in the context of AN. Exercise is generally associated with beneficial effects on mental health, and improved cognitive function and mood [176], which are typically associated with increased BDNF levels [177,178]. However, in patients with AN, this relationship appears to be more complex. Hyperactivity, which is a common feature of AN, may be driven by abnormal BDNF signaling pathways, leading to a paradoxical effect where increased physical activity exacerbates the disorder rather than alleviating it.

The interaction between hyperactivity and BDNF in AN suggests that while exercise might have neuroprotective effects under normal circumstances, the altered BDNF expression observed in AN patients could contribute to the maintenance of pathological behaviors. This complex interplay underscores the need for further research to disentangle these relationships and to develop targeted interventions that address both the physiological and psychological aspects of AN.

### 7.5. Limitations and Future Directions

The paradoxical results observed in circulating BDNF levels in AN patients may also be due to the multiple origins of the molecule. BDNF can cross the blood–brain barrier [179]. However, there is currently no evidence that circulating BDNF corresponds to cerebral BDNF [180,181]. The heterogeneity of cerebral BDNF also call into question the representativeness of circulating BDNF, as it cannot profile the details in different brain regions [108]. Furthermore, due to the different sources of BDNF in peripheral organs, circulating BDNF is unlikely to accurately profile the BDNF levels of a specific organ. Furthermore, plasmatic BDNF has a distinct circadian rhythm, especially in women, which may contribute to the inconsistent results observed in different studies [182].

In conclusion, although research into the role of BDNF as a biomarker for psychiatric disorders and metabolic disorders has been fruitful, the current limitations make it difficult to definitively assert its utility as a diagnostic or prognostic biomarker for AN. Further investigations should include larger patient cohorts with minimized confounding factors, with the aim of establishing a precise correlation between circulating BDNF and BDNF levels in different brain regions and peripheral organs. The use of AN-like animal models may provide valuable insights into the origin and dynamics of circulating BDNF.
